# Evaluation of Retinal Ganglion Cell Layer Thickness in the Early Period After Femtosecond LASIK Surgery

**DOI:** 10.4274/tjo.galenos.2020.29939

**Published:** 2020-08-26

**Authors:** Kemal Özülken, Çağrı İlhan

**Affiliations:** 1 TOBB ETU Medical School, Department of Ophthalmology, Ankara, Turkey; 2 Hatay State Hospital, Clinic of Ophthalmology, Hatay, Turkey

**Keywords:** LASIK, ganglion cell layer, retinal nerve fiber, intraocular pressure, OCT

## Abstract

**Objectives:**

To evaluate the early effects of femtosecond laser-assisted in situ keratomileusis (LASIK) surgery on retinal ganglion cell thickness (GCT), peripapillary retinal nerve fiber thickness (NFT), and central macular thickness (CMT) obtained by spectral domain optical coherence tomography (SD-OCT) in a healthy population.

**Materials and Methods:**

This case-control study included data from the right eye of 40 subjects without any disease other than refractive error and who had undergone femtosecond LASIK surgery. The preoperative, postoperative 1-hour, and postoperative 3-week GCT, NFT, and CMT values obtained by SD-OCT were compared.

**Results:**

The mean age was 27.54±5.99 years (18-45 years). GCT, NFT, and CMT were 18.43±6.03 μm, 107.90±9.01, and 234.3±21.2 μm preoperatively; 18.05±5.93 μm, 108.08±8.92 μm, and 230.1±22.6 μm at postoperative 1 hour; and 17.86±5.27 μm, 107.98±10.13, and 236.3±25.1 μm at postoperative 3 weeks (p=0.159, 0.85, and 0.254, respectively).

**Conclusion:**

There were no changes in GCT, NFT, and CMT values evaluated with SD-OCT in the early period after femtosecond LASIK surgery.

## Introduction

Laser-assisted in situ keratomileusis (LASIK) is the most commonly used method in current refractive surgery practice worldwide.^1^ After lifting corneal tissue, the predetermined amount of tissue in the corneal stroma is ablated by laser energy, and a wide range of refractive errors are corrected. Although it is generally accepted as an effective and reliable method, intraocular pressure (IOP) increases to very high levels even for a short time at the stage of corneal flap formation in both mechanical microkeratome and femtosecond LASIK surgery.^2^ In this regard, the application of LASIK surgery in glaucoma patients or patients with suspected glaucoma is still controversial.^3^

Glaucoma is a progressive optic neuropathy with retinal ganglion cell and retinal nerve fiber damage. High IOP is considered the most important and modifiable risk factor for glaucoma. Progressive disease causes changes in optic disc topography and retinal nerve fiber thickness (NFT).^3^

Optical coherence tomography (OCT) is a noninvasive imaging method that provides real-time in vivo images of the retina.^4^ Spectral domain OCT (SD-OCT) performs 20,000 or more axial scans per second using 840 nm diode laser and provides high resolution imaging with low artifacts.^5^ The quantitative and qualitative analysis of the peripapillary nerve fiber layer obtained by SD-OCT is a highly sensitive diagnostic method that has been used for the detection of glaucomatous damage for many years.^6^ Furthermore, improved SD-OCT software allows for the selective evaluation of internal retinal layers such as the ganglion cell layer and the inner plexiform layer.^7^ The aim of this study was to investigate the effects of femtosecond LASIK surgery on different layers of the retina with SD-OCT images.

## Materials and Methods

Patients who underwent bilateral femtosecond LASIK surgery between August 1, 2018 and March 1, 2019 in the ophthalmology department of TOBB ETU Hospital were included in the study. Approval for the study was obtained from the local ethics committee. Patients were provided detailed information and provided written informed consent before all interventional procedures. The principles in the Declaration of Helsinki were followed at all stages of the study.

Patients with spherical refractive error between -6.00 and +4.0 diopters (D) and/or <3 D cylindrical refractive error, Snellen best corrected visual acuity (BCVA) of at least 20/20 (0.00 logMAR), no known eye disease (such as glaucoma, uveitis, or retinal dystrophies), neurological disease (such as epilepsy or inherited neuropathies), or systemic disease (such as severe vitamin deficiency or metabolic diseases), central corneal thickness (CCT) >500 µm, NFT and central macular thickness (CMT) within normal limits, and normal macular architecture on SD-OCT were included in the study. Patients who failed to meet any of these criteria, those with any known eye or systemic disease, past eye trauma or surgery, history of drug use or dependence, and those who were pregnant or lactating were excluded from the study.

All patients were subjected to a detailed eye examination and asked to discontinue use of soft contact lenses at least 1 week before the operation. Manifest and objective refractive errors, uncorrected visual acuity (UCVA), and BCVA values were obtained. Snellen visual acuity values were converted to logMAR values. IOP was measured with a Goldmann applanation tonometer, and anterior and posterior segment examinations were performed with a biomicroscope. NFT, ganglion cell thickness (GCT), and CMT values were measured without pupillary dilation by SD-OCT (Heidelberg Engineering, Inc., Heidelberg, Germany). CCT and corneal topography were evaluated in detail with Pentacam HR (Oculus, Wetzlar, Germany).

All surgical procedures were performed by an experienced refractive surgeon (K.O.) in a single center. After placement of the lid speculum with topical anesthesia with 0.5% proparacaine (Alcaine, Alcon, Fort Worth, TX, USA) and standard preoperative asepsis protocol, surgery began. The Alcon/WaveLight® FS200 (Alcon Surgical, Fort Worth, TX, USA) was used to create a flap with a thickness of 120 µm and diameter of 9 mm. Following flap lifting and drying of the stromal bed, stromal ablation was performed with Wavelight EX500 (Wavelight, Erlangen, Germany). After the stromal bed was irrigated with a balanced salt solution, the flap was repositioned on the stromal bed, and the operation was terminated. Topical postoperative medication, moxifloxacin 0.5% (Vigamox, Alcon, Fort Worth, TX, USA), was applied 3 times a day for 1 week, along with a prescription for dexamethasone (Maxidex, Alcon, Fort Worth, TX) at decreasing dosages starting with 5 times a day for 3 weeks. Preservative-free artificial tear drops (Refresh, Allergan, Irvine, CA, USA) were added 8 times a day for 2 months. All patients were instructed not to rub their eyes or go swimming for the first month to prevent flap displacement or infectious keratitis.

**Figure 1:**
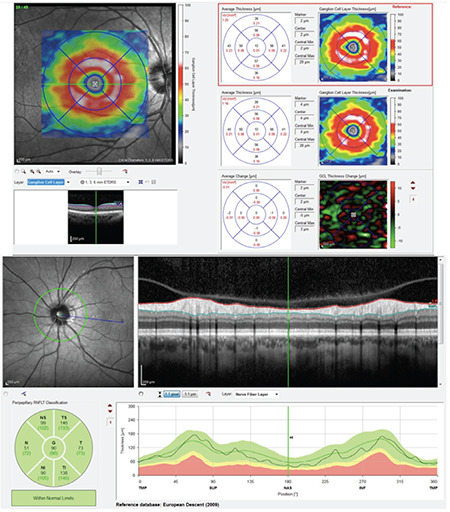
A sample of the ganglion cell and nerve fiber thicknesses report measured by spectral domain optical coherence tomography

SD-OCT was repeated at postoperative 1 hour. If the results were suboptimal, the scan was repeated until reliable results were obtained. Figure 1 demonstrates a sample of GCT and NFT reports measured by SD-OCT. Detailed ophthalmologic examination including manifest refraction, visual acuity, IOP, and anterior and posterior segment examinations was performed at postoperative 1 day, 1 week, and 3 weeks. In addition, SD-OCT evaluation was repeated at postoperative 3 weeks. All follow-up examinations were performed for all subjects.

### Statistical Analysis

The statistical study included data from the right eyes of all patients. The data obtained from the study were analyzed using the Statistical Package for the Social Sciences (SPSS) 24.0 software (IBM Corp., NY, USA). Descriptive statistics were presented as mean ± standard deviation. The normal distribution of the variables was tested using the Kolmogorov-Smirnov test. Since the data did not conform to normal distribution, differences in repeated measures were analyzed using the nonparametric Friedman test. Correlations between variables were investigated using Pearson correlation analysis. Statistical significance was set at p<0.05 for all tests.

## Results

Of the 40 patients included in the study, 18 (45%) were male and 22 (55%) were female. The mean age of the patients was 27.54±5.99 years (range 18 to 45 years). The mean spherical equivalent of the patients preoperatively was -2.13±1.82 D (+2.73 to 5.25 D) and at postoperative 3 weeks was -0.23±0.18 D (+0.45 to 0.38 D). The preoperative mean UCVA was 0.84±0.22 logMAR (+1.60 to 0.30 logMAR), while it was 0.11±0.04 logMAR (0.20 to -0.10 logMAR) at postoperative 3 weeks. Table 1 summarizes the preoperative and postoperative third week clinical characteristics of the subjects.

According to the results of the SD-OCT evaluation, preoperative mean GCT was 18.43±6.03 µm (11 to 32 µm), and this value was 18.05±5.93 µm (10 to 31 µm) at postoperative 1 hour and 17.86±5.27µm (11 to 32 µm) at postoperative 3 weeks (p=0.159). The mean peripapillary global NFT were 107.90±9.01 µm (90 to 134 µm) preoperatively, 108.08±8.92 µm (88 to 131 µm) at postoperative 1 hour, and 107.98±10.13 µm (91 to 135 µm) at postoperative 3 weeks (p=0.851).

**Figure 2:**
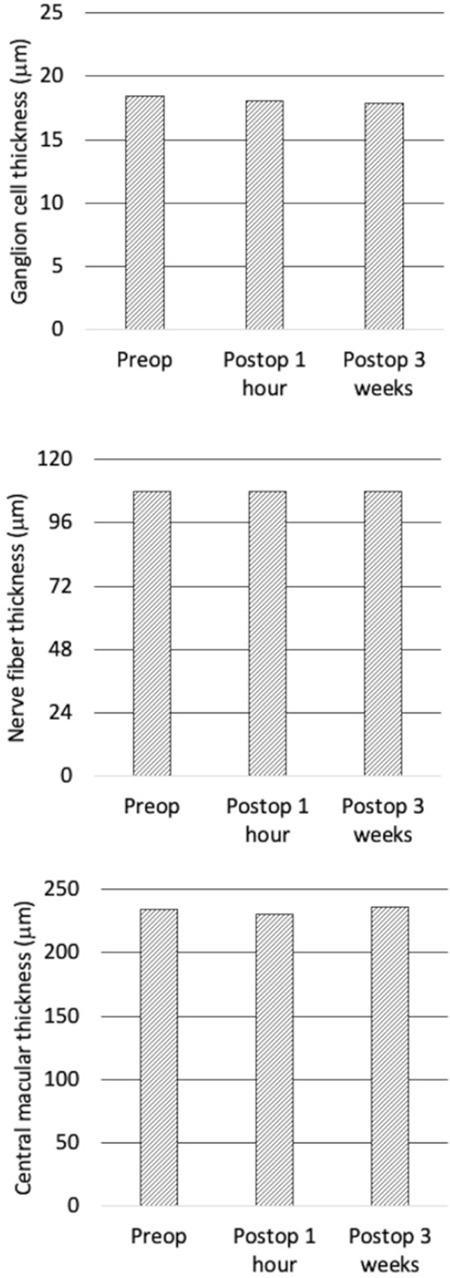
Demonstration of ganglion cell, nerve fiber, and central macular thickness measurements obtained preoperatively (preop) and at postoperative (postop) 1 hour and 3 weeks

Similarly, while preoperative CMT was 234.3±21.2 µm (212 to 255 µm), this value was 230.1±22.6 µm (207 to 253 µm) at postoperative 1 hour and 236.3±25.1 µm (205 to 267 µm) at postoperative 3 weeks (p=0.254) (Table 2). There were significant positive correlations between GCT, NFT, and CMT values preoperatively, at postoperative 1 hour, and postoperative 3 weeks (all p values were <0.001) (Table 3). Figure 2 demonstrates preoperative, postoperative 1-hour, and postoperative 3-week GCT, NFT, and CMT values (Figure 2).

## Discussion

During LASIK surgery, IOP is known to be at very high levels for a period of time. Hernandez-Verdejo et al.^2^ reported in their experimental studies that in the LASIK surgery performed with mechanical microkeratome, IOP increased to 122.5 mmHg and 160.5 mmHg for a mean of 21.4 s and 15 s in the suctioning and cutting phases, respectively. In femtosecond LASIK surgery, these values were found to be 89.2 mmHg and 119.3 mmHg for 40 s and 52.8 s, respectively.^2^ The structural and functional effects of the IOP value above the normal value in both microkeratome and femtosecond LASIK surgery are not yet fully known. Therefore, recent studies have aimed to address concerns about the safety of LASIK surgery in glaucoma patients or in patients with suspected glaucoma.

Computer-aided imaging methods are efficient, reliable, and objective and have an important role in the follow-up of glaucomatous patients. Among these, OCT is used more frequently than other methods. OCT allows direct structural evaluation by direct thickness measurement using the reflection of a near-infrared beam from the posterior segment structures.^8^  In recent years, many studies with OCT have shown that there is no significant change in NFT after both mechanical microkeratome and femtosecond LASIK surgery.^9,10,11,12^ Although it has been reported that evaluation of the ganglion cell complex (nerve fiber, ganglion cell, and inner plexiform layers in the macular area) in glaucomatous patients does not contribute to the evaluation of peripapillary NFT, the investigation of GCT after LASIK surgery is a newer approach.^13,14^

Zivkovic et al.^15^ reported no significant changes in the thickness of the ganglion cell–inner plexiform layer after microkeratome-assisted LASIK surgery. Zhang et al.^7^ reported no significant change in the thickness of the nerve fiber layer and ganglion cell complex after femtosecond LASIK surgery. In the present study, which evaluated GCT with SD-OCT in the early period after femtosecond LASIK surgery, no significant change was observed in GCT and NFT values, similar to the literature, and the results correlated with CMT.

In another study, Katsanos et al.^16^ examined NFT after laser-assisted subepithelial keratomileusis and femtosecond LASIK surgery. In contrast to our study, they found that temporal-inferior and average NFT values were significantly higher after femtosecond LASIK than the preoperative values. Moreover, they suggested that LASIK-induced corneal changes might have affected postoperative image acquisition. They also stated that they could not exclude increased postoperative NFT as a sign of axonal edema.

In a study conducted in 102 eyes treated with a Ziemer LDV femtosecond laser (Ziemer Group, Port, Switzerland) and 102 eyes treated with an M2 microkeratome, Zhang and Zhou^17^ showed that IOP elevations observed during LASIK may lead to retinal edema in the postoperative period by preventing ocular blood flow and axial transport. They used Fourier-domain OCT to assess macular, ganglion cell complex and nerve fiber layer thicknesses and found that the mean foveal and parafoveal NFT values increased in both groups at 30 minutes after LASIK compared to preoperative values. In addition, they observed that the difference in NFT measurements disappeared on the first postoperative day and remained statistically similar during the 1-year follow-up compared to preoperative values.

Similarly, in this study, SD-OCT measurements were performed 1 hour after the procedure to investigate the changes in NFT and GCT in the short term after femtosecond LASIK surgery, and no statistical difference between preoperative and postoperative measurements was found. This may be due to the fact that the OCT device used in this study is different. In addition, the time between the mean vacuum on and vacuum off sets generated by the different laser platforms and the increase in the maximum IOP values during the procedure may affect postoperative outcomes. The relatively short time between the average vacuum on/vacuum off sets generated by the new generation femtosecond laser platform used in this study could be the reason there was no increase in the NFT and GCT in this study.

Despite the results of this study that indicate no change in the GCT, NFT, and CMT measured by the SD-OCT in the early period, it cannot be concluded that femtosecond LASIK surgery is completely safe in subjects with glaucoma or suspected glaucoma. In this regard, every subject who is a candidate for refractive surgery should be evaluated in detail and glaucoma risk factors identified because this surgery may change corneal viscoelastic properties, corneal permeability, and topical antiglaucoma medication response.^18^ Further studies evaluating the long-term effects of femtosecond LASIK surgery with novel diagnostic tools may help to overcome these diagnostic and management challenges in patients with confirmed or suspected glaucoma.

### Study Limitations

Strengths of this study are that we used a new-generation SD-OCT device with high sensitivity and resolution and included a relatively homogeneous sample. However, the small number of patients, short follow-up period, and absence of SD-OCT measurements on the postoperative first day may be considered limitations.

## Conclusion

In conclusion, there was no change in GCT, NFT, and CMT measured by the SD-OCT in the early period after femtosecond LASIK surgery.

## Figures and Tables

**Table 1:**
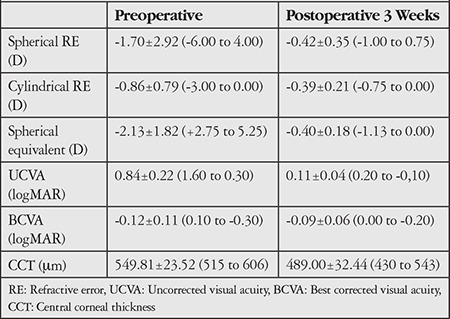
Clinical characteristics of the subjects at preoperative and postoperative 3 weeks

**Table 2:**
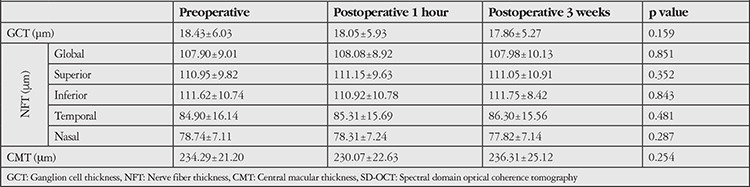
GCT, NFT, and CMT values measured by SD-OCT

**Table 3:**
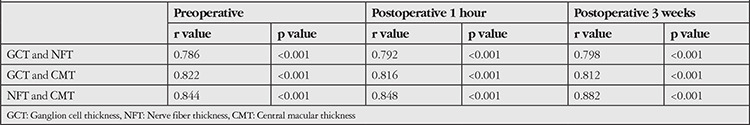
Details in correlation between GCT, NFT, and CMT values preoperatively, at postoperative 1 hour, and at postoperative 3 weeks.
